# Clinicopathological characteristics and predictors of poor outcome in anti-glomerular basement membrane disease – a fifteen year single center experience

**DOI:** 10.1080/0886022X.2020.1854301

**Published:** 2020-12-17

**Authors:** Zafirah Zahir, Asif Sadiq Wani, Narayan Prasad, Manoj Jain

**Affiliations:** a PDCC Renal Pathology, Sanjay Gandhi Post Graduate Institute of Medical Sciences, Lucknow, India; b Department of Nephrology, Sanjay Gandhi Post Graduate Institute of Medical Sciences, Lucknow, India; c Department of Nephrology and Renal Transplantation, Sanjay Gandhi Post Graduate Institute of Medical Sciences, Lucknow, India; d Department of Pathology, Sanjay Gandhi Post Graduate Institute of Medical Sciences, Lucknow, India

**Keywords:** Anti-glomerular basement membrane disease, atypical anti-glomerular basement membrane disease, double antibody positive anti-glomerular basement membrane disease, glomerulonephritis, pulmonary renal syndrome, rapidly progressive glomerulonephritis

## Abstract

**Introduction:**

Anti-glomerular basement membrane (anti-GBM) disease is a small vessel vasculitis affecting the renal and lung capillary beds. We aim to study the clinicopathological characteristics and predictors of poor outcome of this disease in our population.

**Materials and methods:**

This is a 15 year retrospective, single center observational study of Indian cohort. Patients with biopsy proven anti-GBM disease were studied.

**Results:**

Anti-GBM disease was found in 0.5% of the total cases. The mean age at presentation was 46.7 years. Compared to renal limited disease those with pulmonary-renal syndrome had a higher frequency of hypertension, oliguria, percentage of crescents, interstitial inflammation and glomerulosclerosis. Double positive (anti-GBM and ANCA antibodies) patients showed more of glomerulosclerosis, tubular atrophy/interstitial fibrosis (IFTA) as well as periglomerular granulomas on biopsy. Patient survival at one year was 40.4% and death censored renal survival was 9.7%. Factors affecting the dialysis dependency at presentation were oligoanuria (*p* = .04), creatinine levels >5.7 mg/dl (*p* = .003), and high mean anti-GBM titers (*p* = .008). Atypical cases accounted for 8.3% of these patients. Oligoanuria (HR = 5.0, *p* = .05), high serum creatinine (HR = 1.55, *p* = .05), severe glomerulosclerosis (HR = 1.09, *p* = .03), and IFTA (HR = 2, *p* = .04) were associated with poor renal outcome. Advanced age (HR = 1.92, *p* = .03), high serum creatinine (HR = 1.9, *p* = .04) and high anti-GBM titers (HR = 1.01, *p* = .03) were associated with poor patient survival.

**Conclusions:**

Anti-GBM is a rare disease with poor prognosis and varied presentations. Patients with pulmonary-renal syndrome showed severe disease whereas double positive had more of chronic changes. The predictors of poor prognosis include advanced age, oliguria, serum anti-GBM levels, serum creatinine levels, degree of glomerulosclerosis and IFTA. Atypical anti-GBM cases should be kept in mind while evaluating renal biopsies.

## Introduction

Anti–glomerular basement membrane (anti-GBM) disease is a small vessel vasculitis affecting the renal and lung capillary beds [1] with an incidence of 1–2 cases/million population/year [2]. The majority of patients present with features of rapidly progressive glomerulonephritis (RPGN) and have widespread glomerular crescent formation on kidney biopsy [1]. Coexisting pulmonary involvement is varied ranging from as low as 23% to as high as 63%. When both lungs and kidneys are involved it is known as pulmonary-renal syndrome [3]. The pathogenesis of anti-GBM Glomerulonephritis is the presence of circulating IgG autoantibodies against type IV collagen which are visualized as linear IgG deposits along Glomerular Basement membrane (GBM) [4]. Treatment is directed toward the removal of pathogenic autoantibodies, with the use of plasma exchange, along with corticosteroids and cytotoxic therapy to hault the ongoing autoantibody production and tissue damage [1]. The prognosis of these patients remains dismal [5], even after aggressive treatment with a combination of plasma exchange, corticosteroids, and immunosuppressive agents [6]. With more than sixty years into this disease, there is still a lack of complete understanding probably due to rarity of the disease and its varied presentations. The presentation of anti-GBM glomerulonephritis along with other kidney diseases like ANCA-associated vasculitis and membranous nephropathy occurs at higher rates than would be expected by chance alone and also its atypical presentations are increasingly reported [1]. We studied the clinico-pathological correlates of anti-GBM disease and predictors of poor outcome. We also studied characteristics of dual positive and atypical anti-GBM variants.

## Materials and methods

### Study design, setting and participants

This is a 15 year retrospective, single-center cohort, observational study (July 2004–July 2019) conducted at Sanjay Gandhi Post-Graduate Institute of Medical Sciences, India. Patients with biopsy proven anti-GBM glomerulonephritis (anti-GBM GN) with or without positive serology were included in the study. The term ‘anti-GBM GN’ was used while referring specifically to the kidney involvement and ‘anti-GBM disease’ while referring to the broader spectrum of renal and alveolar damage

***Exclusion criteria (bias)*:** Patients with other coexisting kidney diseases were excluded except for those having concomitant ANCA positivity (dual positive cases) or coming under the spectrum of atypical anti-GBM disease.

***Renal biopsy evaluation*:** Two core biopsies, taken under ultrasound guidance by biopsy gun, one for light microscopy and immunofluorescence each, were evaluated by either of the two nephropathologists. For light microscopy, standard sections were cut from renal biopsy received in 10% buffered formalin. Biopsies were evaluated for endocapillary, extracapillary or mesangial proliferation, fibrinoid necrosis, interstitial inflammation, percentage of glomerulosclerosis, interstitial fibrosis, and tubular atrophy. For imunofluorescence, the biopsy was transported in normal saline. Standard sections were cut in cryostat at a temperature of −20 °C. The percentage of sclerosed glomeruli were graded as Grade 1(<25%), Grade 2 (25–50%), and Grade 3 (>50%). Interstitial fibrosis and tubular atrophy were graded as mild (<25%), moderate (25–50%), and severe (>50%). Interstitial inflammation was also graded as mild (<25%), moderate (25–50%), and severe (>50%).

### Data source and variables

Clinical features, laboratory data, histopathology and follow up records of the patients were retrieved from the hospital information system. Results on immunofluorescence were noted from the pathology reports. Anti-GBM antibodies were measured using enzyme linked immunoassay (ELISA) and serum creatinine was measured by enzymatic method. The treatment received by the patients was recorded from treatment charts.

### Definitions

Patients <18 years of age were regarded as children. Microscopic hematuria was defined as at least 5 red cells per high-power field on microscopic examination or positive blood by urine dipstick. Nephrotic syndrome was defined as nephrotic range proteinuria of >3.5 g per 24 h per 1.73 m^2^ (in children, >40 mg/m^2^/hr or PCR >2000 mg/g [>200 mg/mmol] or >300 mg/dl or 3 + on urine dipstick) along with hypoalbuminemia and edema [7,8]. Rapidly progressive glomerulonephritis (RPGN) was defined as the rapid loss of renal function within days to weeks, accompanied by nephritic syndrome features (proteinuria, glomerular hematuria and often oliguria) [9]. Oliguria was defined as urinary output of <400 mL/24 h while anuria was defined as urinary output of <100 mL/24 h. Advanced renal failure at presentation was defined as serum creatinine >5.7 mg/dl, in accordance with a few previous studies [4]. Dialysis-dependent renal failure was defined as the need to dialyze the patient within 72 h of admission to the hospital [6]. A diagnosis of pulmonary hemorrhage was rendered in patients with overt hemoptysis and/or pulmonary interstitial opacities on computed tomography (CT) chest and/or bronchoalveolar lavage showing alveolar hemorrhage [10].

### Atypical anti-gbm

This was defined as those cases of anti-GBM disease that had atypical clinical course (like an indolent course, no pulmonary involvement, and undetectable circulating anti-GBM antibody) and/or atypical histological findings like absent or focal crescents, monoclonal antibody-associated anti-GBM disease or anti GBM disease with IgA nephropathy [11].

### Study outcomes

The primary outcome of interest was renal and patient survival at one year. ESRD was defined as a decline in the patient's kidney function to a level at which either long-term dialysis or kidney transplantation is required to sustain life [12].

Secondary Outcomes studied were complete and partial remission. Complete remission (CR) was defined as the return of serum creatinine to the previous baseline, plus the reduction in proteinuria to <0.5 g/day or 0.5 g/g creatinine by urinary proteinuria: creatinine ratio (uPCR). Partial remission (PR) was defined as stable (±25%) or improved serum creatinine, but not to normal, plus ≥50% reduction in proteinuria to <3 g/day (or 3 g/g uPCR) [7]. eGFR was calculated using the CKD-EPI equation [13].

### Statistical analysis

The statistical analysis was done using SPSS Version 23.0. (IBM Corp., Armonk, NY, USA). Categorical variables, expressed as numbers and percentages, were compared using Chi Square and Fisher's exact test, as applicable. Continuous variables, reported as mean or median (depending on the normality of data) were compared using t test, one-way ANOVA, Wilcoxon rank-sum methods as appropriate. Renal survival and Patient survival was determined using the Kaplan–Meier method and group comparisons for survival was performed using the log-rank test. Cox Regression univariate and multivariate analyses were used to assess the factors affecting renal and patient analysis. The patients who were lost to follow up were removed from survival analysis. *p* Value was taken as significant when it was ≤ .05 [14].

**Ethics approval and consent:** This was not sought as it was a retrospective study and did not involve any active participation of the patients. The patients whose data were analyzed could also not be contacted further. Many patients were de-identified and waiver of their consent did not adversely affect the rights and welfare of the participants. A local ethics committee ruled that no formal ethics approval was required in this particular case.

## Results

### Patient characteristics

A total of 9080 biopsies were studied in 15 years (July 2004–July 2019) of which 51 were provisionally included under anti-GBM glomerulonephritis. Three patients for whom immunofluorescence reports were unavailable were excluded from the study. Almost all patients presented with rapidly progressive glomerulonephritis (RPGN). The mean age of patients was 46.7 years and male: female ratio was 1.1:1. The demographic profile and laboratory parameters of these patients are shown in Table 1.

**Table 1.** Showing demographic profile, laboratory parameters and biopsy findings of patients with anti-glomerular basement membrane disease (*n* = 48).

Age (Years)	46.7 ± 17
M:F	1.1:1
Comorbidities	30 (62.5%)
Hypertension	28 (58.3%)
Diabetes	3 (6.2%)
Lung involvement	14 (29.2%)
Normal anti-GBM Titers	2 (4.16%)
ANCA positivity	14 (29.2%)
MPO	11 (78.5%)
PR3	03 (21.4%)
ANA positivity	9 (18.7%)
^a^Oligoanuria	28 (58.3%)
Mean Creatinine (mg/dl)	8.7 ± 3.3
^b^Anemia	40 (83.3%)
^c^Leukocytosis	12 (25%)
^d^Thrombocytopenia	10 (20.8%)
Nephrotic Range Proteinuria	15 (31.3%)
^e^Low Complements	6 (12.5%)
Dialysis dependency at presentation	38 (79.2%)

ANCA: Anti-neutrophil cytoplasmic antibodies; MPO: myeloperoxidase; PR3: proteinase 3.

^a^Oliguria was defined as urinary output of <400 mL/24 h while anuria was defined as urinary output of <100 mL/24 h.

^b^Anemia was defined as per WHO criteria of hemoglobin <13mg/dl in males, <11mg/dl in females and <12mg/dl in children.

^c^Leukocytosis was defined as leukocytes >10.5 x 10^9^/L.

^d^Thrombocytopenia was defined as platelet counts <150 x 10^9^/L.

^e^The Hospital Reference Laboratory normal range for C3 was 80–178 mg/dl and C4 was 12–42mg/dl.

**Renal biopsy findings**: The most common morphological pattern was crescentic glomerulonephritis, occurring in 44 (91.6%) patients. Two patients had <50% crescents in their biopsy. The mean percentage of crescents on biopsy was 76.5%±22.4%. Most of the patients had cellular crescents, the mean percentage of cellular crescents being 65.4%. In addition, mesangial hypercellularity was noted in 16.7% and endocapillary proliferation was seen in 10.4% of the cases. Mild glomerulosclerosis was seen in 16 patients, 12 had moderate and 8 had severe glomerulosclerosis. Interstitial fibrosis and tubular atrophy were seen in 43 patients, mild in 19, moderate in 14 and severe in 10 patients. On immunofluorescence all of the patients had a linear staining for IgG along GBM. Varying amounts of interstitial inflammation was seen- mild in 18 patients, moderate in 21 patients and severe in 9 patients with formation of periglomerular granulomas in 6 patients.

## Anti-GBM variants

### Pulmonary-renal syndrome

Coexisting lung involvement was present in 14 (29.2%) patients. The mean age of patients with lung involvement was 47.7 ± 17.3 years and 46.3 ± 17.6 years in patients without lung involvement. Hypertension was seen in only 5 (35.7%) patients without lung involvement whereas it was noted in 23 (67.6%) patients showing lung involvement. The mean serum creatinine of renal limited disease patients was 8.05 mg/dl while it was 9.02 mg/dl in patients with pulmonary-renal syndrome. Those patients with coexisting lung involvement showed higher frequency of oligoanuria [11 (78.5%) vs 17 (50%)], mean percentage of crescents [84.45% vs 73.3%], formation of periglomerular granulomas [4 (28.5%) vs 2 (5.8%)] as well as dialysis dependency at presentation [13 (92.8%) vs 25 (73.5%)] as compared to those with renal limited disease. The differences between the patients with and without lung involvement are shown in Table 2.

**Table 2.** Differences between patients with and without lung involvement.

	Without lung involvement (*n* = 34)	With lung involvement (*n* = 14)	*p* Value
M:F	1.1:1	1.3:1	
Mean Age	46.3 ± 17.6	47.7 ± 17.3	.75
Hypertension	23 (67.6%)	5 (35.7%)	.04
^a^Oligoanuria	17 (50%)	11 (78.5%)	.06
Mean Creatinine(mg/dl)	8.05	9.02	.02
^b^Anemia	32 (94.1%)	14 (100%)	.20
^c^Leukocytosis	7 (20.5%)	5 (35.7%)	.21
^d^Thrombocytopenia	6 (17.6%)	4 (28.5%)	.32
Mean Crescent Percentage	73.3%	84.45%	.06
Glomerulosclerosis			
MILD	11 (32.3%)	05 (35.7%)	.20
MODERATE	10 (29.4%)	02 (14.2%)	
SEVERE	07 (20.5%)	01 (7.1%)	
IFTA			
MILD	13 (38.2%)	06 (42.8%)	.86
MODERATE	11 (32.3%)	03 (21.4%)	
SEVERE	07 (20.5%)	03 (21.4%)	
Interstitial Inflammation (Periglomerular Granulomas)	2 (5.8%)	4 (28.5%)	.03
Mean Anti-GBM Titers (IU/ML)	94.3	112.8	.24
Nephrotic Range Protieinuria	13 (38.2%)	2 (14.2%)	.09
Dialysis Dependency At Presentation	25 (73.5%)	13 (92.8%)	.04
Outcome (CR)	4 (11.7%)	1 (7.14%)	.06

IFTA: Interstitial Fibrosis and Tubular Atrophy; CR: Complete Remission.

^a^Oliguria was defined as urinary output of <400 mL/24 h while anuria was defined as urinary output of <100 mL/24 h.

^b^Anemia was defined as per WHO criteria of hemoglobin <13mg/dl in males, <11mg/dl in females and <12mg/dl in children.

^c^Leukocytosis was defined as leukocytes >10.5 x 10^9^/L.

^d^Thrombocytopenia was defined as platelet counts <150 x 10^9^/L.

### Double positive disease

Fourteen (29.2%) patients had double positive disease (anti-GBM and ANCA associated glomerulonephritis). The mean age of the patients with single and double antibody positive disease was similar (47.6 ± 17.7 and 44.7 ± 16.9 respectively) in our study. Those patients with double positive antibody showed a higher percentage of glomerulosclerosis (85.7% vs 70.5%) and severe interstitial fibrosis and tubular atrophy [6 (42.8%) vs 4 (11.7%)] as compared to single anti-GBM antibody positive group. All six patients which had periglomerular granulomas belonged to the double antibody positive group. The mean anti-GBM antibody titers were higher in single anti-GBM antibody positive group as patients as compared to double antibody positive patients (110.5 IU/ml vs 74.7 IU/ml). The clinico-pathological differences between the single and double antibody-positive patients are shown in Table 3.

**Table 3.** Clinicopathological differences between dual and single antibody positive patients.

	Single positive (*n* = 34)	Double positive (*n* = 14)	*p* Value
M:F	1.4:1	1:1.3	
Hypertension	20 (58.8%)	8 (57.1%)	.81
Mean Age	47.6 ± 17.7	44.7 ± 16.9	.52
^a^Oliguria/Anuria	17 (50%)	11 (78.5%)	.06
Mean Creatinine (mg/dl)	8.8 ± 3.5	8.4 ± 2.9	.41
^b^Anemia	32 (94.1%)	14 (100%)	.40
^c^Leukocytosis	8 (23.5%)	4 (28.5%)	.72
^d^Thrombocytopenia	7 (20.5%)	3 (21.4%)	.90
Fibrocellular Crescent	43.14%	29.41%	.05
Mean Percentage of Crescents (%)	84.5 ± 13.4	73.2 ± 24.7	.08
Glomerulosclerosis			
MILD	10 (29.4%)	6 (42.8%)	.31
MODERATE	7 (20.5%)	5 (35.7%)	
SEVERE	7 (20.5%)	1 (7.1%)	
IFTA			
MILD	17 (50%)	2 (28.5%)	.01
MODERATE	8 (23.5%)	6 (42.8%)	
SEVERE	4 (11.7%)	6 (42.8%)	
Interstitial Inflammation (Periglomerular Granulomas)	0 (0%)	6 (42.8%)	.001
Mean anti-GBM Antibody Titers (IU/ml)	110.5 ± 82	74.7 ± 33.5	.04
Nephrotic Range Proteinuria	10 (29.4%)	5 (35.7%)	.72
Dialysis Dependency at Presentation	27 (79.4%)	11 (78.5%)	.60
Outcome(CR)	3 (8.8%)	2 (14.2%)	.05

IFTA: Interstitial Fibrosis and Tubular Atrophy; CR: Complete Remission.

^a^Oliguria was defined as urinary output of <400 mL/24 h while anuria was defined as urinary output of <100 mL/24 h.

^b^Anemia was defined as per WHO criteria of hemoglobin <13mg/dl in males, <11mg/dl in females and <12mg/dl in children.

^c^Leukocytosis was defined as leukocytes >10.5 x 10^9^/L.

^d^Thrombocytopenia was defined as platelet counts <150 x 10^9^/L.

### Atypical anti-GBM cases

Four patients with atypical features were noted in our study accounting for 8.3% of the total cases. Two patients had coexisting IgA nephropathy, third one had membranoproliferative glomerulonephritis (MPGN) picture with no crescent formation and the fourth had tubulo-interstitial nephritis histology on biopsy. The details of these patients are shown in Table 4. On comparison of these cases, atypical ones showed lower frequencies of renal pulmonary syndrome (p .05) and oliganuria (p .03), had lower mean serum creatinine (p .003) and anti-GBM titers (p .05), as well as lesser degree of IFTA (.01) on biopsy as compared to the classical anti-GBM cases.

**Table 4.** Clinicopathological characteristics of atypical anti- glomerular basement membrane cases.

Parameter	Case 1	Case 2	Case 3	Case 4
Age	37	34	47	69
Gender	Female	Male	Male	Male
S. Creatinine (mg/dl)	9.9	2.1	5.7	9
Clinical Presentation	Advanced Renal Failure	Nephrito-Nephrotic Picture	Nephrito-Nephrotic Picture	Advanced Renal Failure
Diabetic	No	No	No	No
Oligoanuria	Absent	Absent	Absent	Absent
Hematuria	4+	3+	4+	4+
Proteinuria	3+	4+	3+	3+
Anti-GBM Titers (IU/ml)	31.6	38.3	64.5	156
ANCA	Negative	Negative	Negative	Negative
Complements	Normal	Normal	Normal	Low
ANA	Negative	Negative	Negative	Negative
Anti-dsDNA	Negative	Negative	Not Done	Negative
Lung Involvement	No	No	No	Yes
Histomorphology	Crescentic Glomerulonephritis	Membrano-proliferative glomerulonephritis (no crescent)	Crescentic Glomerulonephritis with Mesangial proliferation	Tubulo-interstitial nephritis Unremarkable glomeruli
Immunofluorescence IgG	4+ Linear along GBM	3+ Linear along GBM	2+ Linear along GBM	4+ Linear along GBM
IgM	Negative	3+ Linear along GBM	Negative	Negative
IgA	4+ Granular Mesangial	2+ Linear along GBM	3+ Granular Mesangial	Negative
C3	4+ Linear along GBM	2+ Linear along GBM	3+ Linear along GBM	3+ Linear along GBM
C1q	Negative	3+ Linear along GBM	Negative	Negative
Outcome	ESRD	CR	CR	CR

GBM: Glomerular Basement Membrane; ESRD: End Stage Renal Disease; CR: Complete Remission.

## Treatment and outcome

Data on treatment were available in 43 patients only. Five patients did not have any treatment records due to lost to follow up, early discharge, early death of the patient or incomplete data available on treatment charts. Immunosuppression was given in 31 patients with intense (triple) therapy comprising of plasma exchanges (seven), corticosteroid, and cyclophosphamide/mycophenolate mofetil in 21 patients. Five patients received plasmapheresis along with steroids only and cyclophosphamide was not given or withdrawn early due to cytopenias. Five patients did not receive plasmapheresis as either it was not tolerated or not affordable to the patient. Remaining twelve patients did not receive any immunosupression because of the following reasons- advanced renal failure with 100% cellular crescents, concomitant severe infections or therapy related complications.

Thirty eight patients were dialysis dependent at presentation. Factors affecting the dialysis dependency at presentation were oligoanuria (*p* = .04), creatinine levels >5.7 mg/dl (*p* = .003), and high mean anti-GBM titers (*p* = .008).

**Figure 1.**
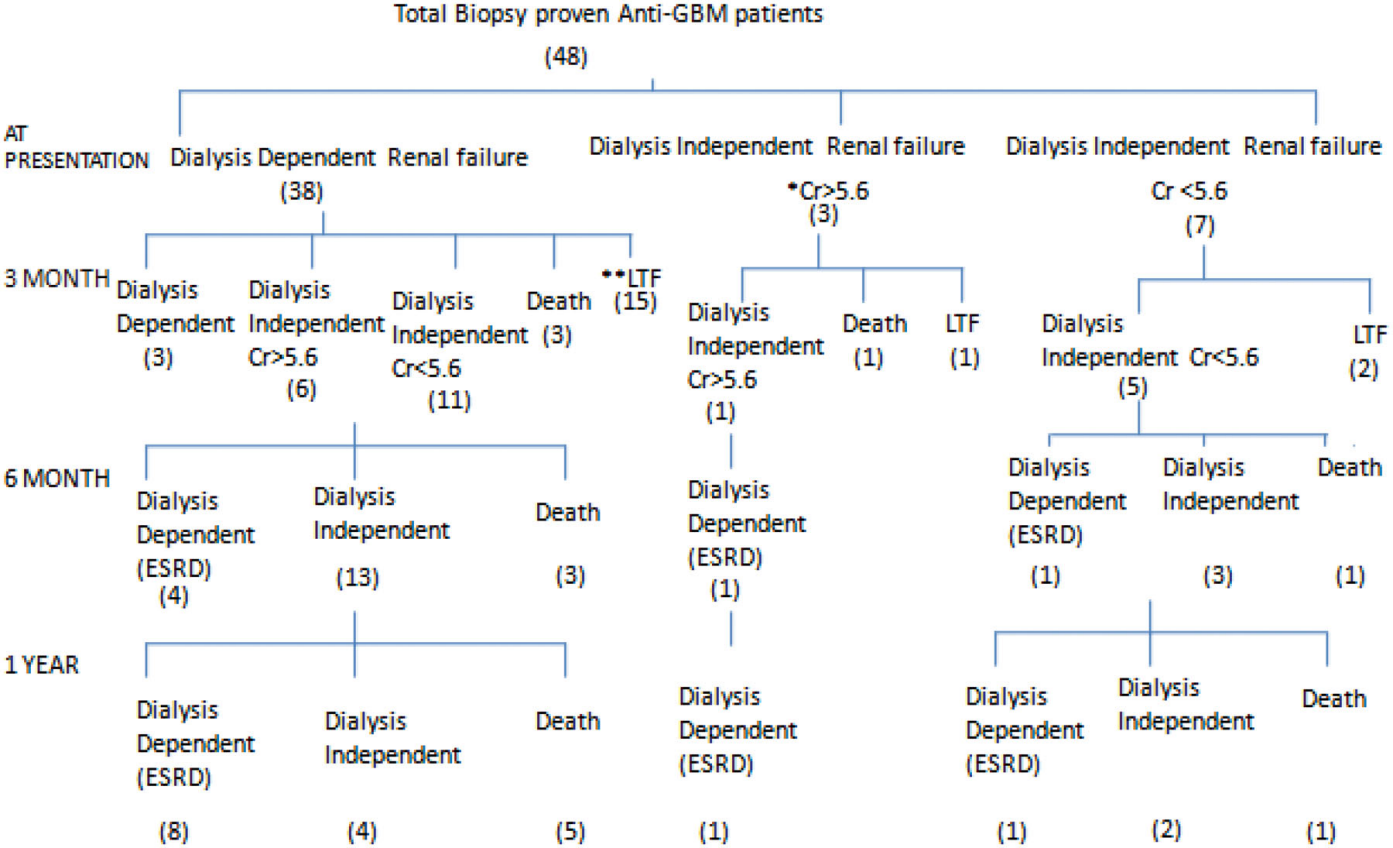
Flow chart of anti- glomerular basement mmembrane patients showing the follow-up results of patients at 3 months, 6 months and one year. *Cr: Serum Creatinine, **LTF: Lost to follow up.

**Figure 2.**
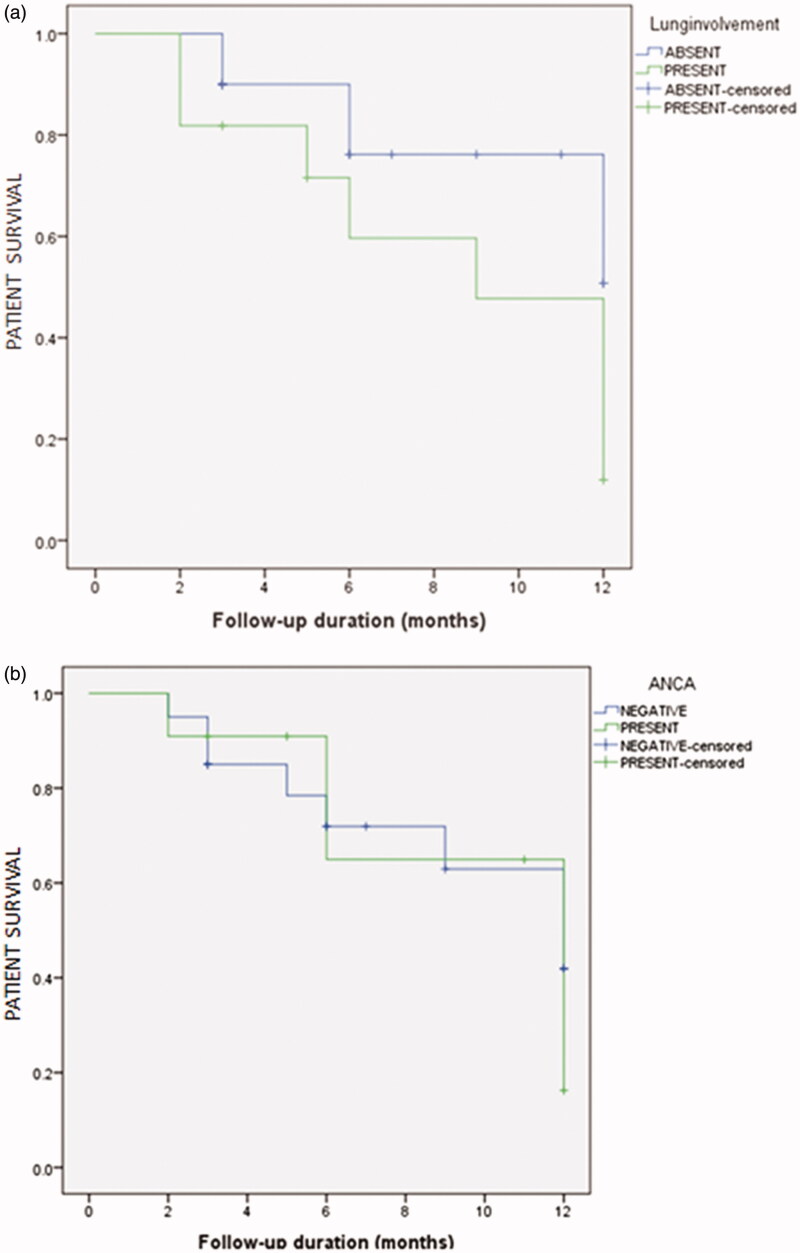
Kaplan-Meier Curve for Anti-GBM disease showing similar patient survival for (a) Patients with coexisting lung involvement (*p* = .06). (b) Patients with dual-antibody positive (*p* = .6).

**Figure 3.**
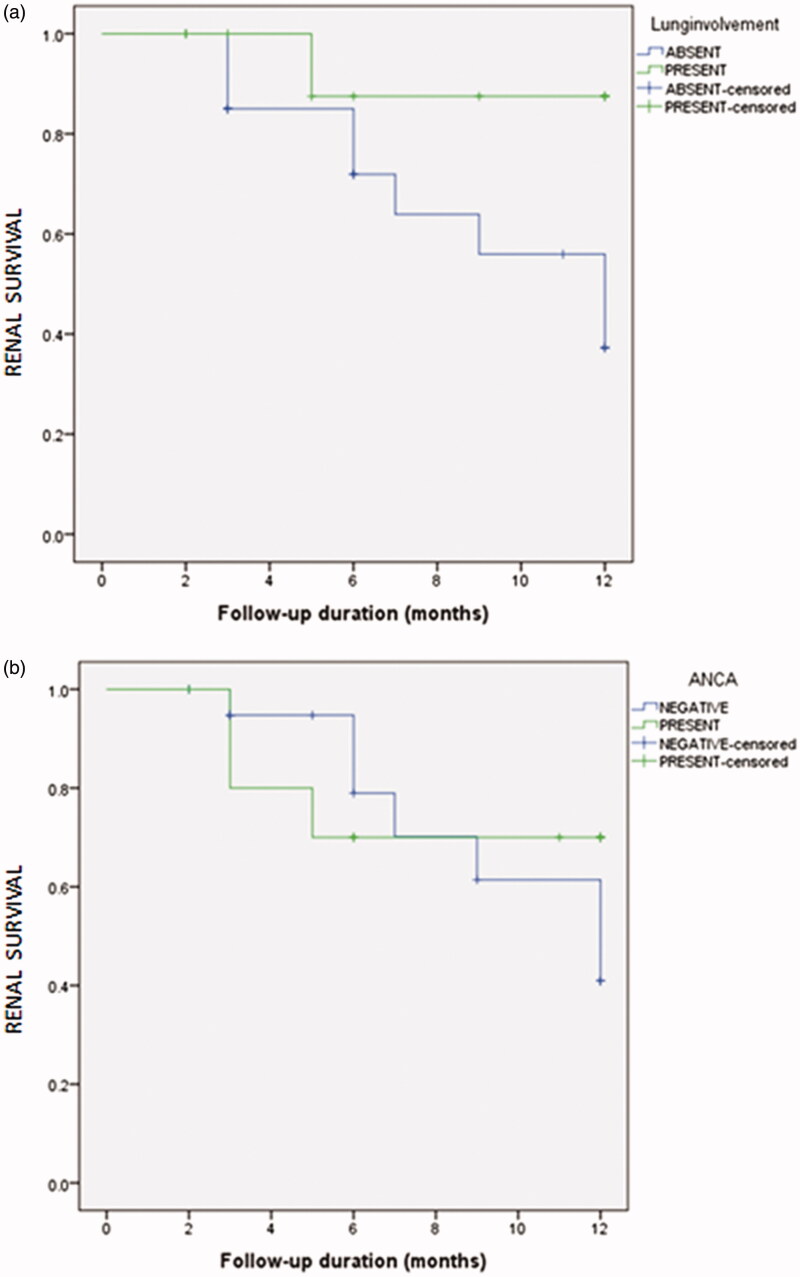
Kaplan-Meier Curve for Anti-GBM disease showing (a) Non-significant difference in the renal survival for patients with coexisting lung involvement (*p* = .08) and (b) similar renal survival for patients with dual-antibody positive (*p* = .6).

The median follow-up period was 7 (IQR 9) months. A flow chart of patients showing the follow-up results of patients at 3 months, 6 months and one year is shown in Figure 1. Out of 48 patients 17 were lost to follow up. Fourteen patients died during one year and 10 developed ESRD among the surviving patients. Patient survival at one year was 40.4% (95%CI 18.5%–62.3%). Death censored renal survival was 9.7% (95% CI 8.4%–11.1%) at one year. The patient survival and renal survival comparisons between the patients with renal limited disease and pulmonary renal syndrome are shown in Figures 2(a) and 3(a). The patient survival and renal survival comparisons between the patients with single antibody and double antibody disease are shown in Figures 2(b) and 3(b).

On univariate analysis oliganuria (HR = 1.5, *p*=.002), high serum creatinine (HR = 1.5, *p* = .05), severe glomerulosclerosis (HR = 1.003, *p* = .003), severe IFTA (HR = 1.4, *p* = .03) and coexisting pulmonary hemorrhage (HR = 1.07, *p* = .01) were associated with ESRD at one year where as in multivariate analysis oliganuria (HR = 5.1, *p* = .05), high serum creatinine (HR = 1.55, *p* = .05), severe glomerulosclerosis (HR = 1.09, *p* = .03) and severe IFTA (HR = 2, *p* = .04) were associated with poor renal survival (Table 5).

**Table 5.** Showing prognostic factors for renal survival at one year in anti- glomerular basement membrane patients.

	Univariate analysis			Multivariate analysis		
Variable	HR	CI (±95%)	*p* Value	HR	CI (±95%)	*p* Value
Age (Advanced)	1.00	0.96–1.05	.71	1.09	0.98–1.21	.09
Oligoanuria	1.5	1.37–6.2	.002	5.1	1.31–8.72	.05
S. Creatinine (mg/dl)	1.5	1.09–2.62	.05	1.55	1.30–2.01	.05
Percentage of Crescents	0.98	0.95–1.01	.21	1.01	0.95–1.07	.60
^a^Severe Glomerulosclerosis	1.003	0.97–1.03	.003	1.09	1.009–1.81	.03
^b^Severe IFTA	1.42	1.24–3.27	.03	2	1.21–3.12	.04
High Anti-GBM Titers (per increase by 20 IU/ml)	0.99	0.98–1.00	.08	0.98	0.97–1.00	.07
Dialysis Dependency At Presentation	0.19	0.02–1.53	.11	2.02	0.08–50	.66
ANCA Positivity	1.02	0.24–4.30	.91	1.09	0.01–0.74	.96
Lung Involvement	1.07	1.05–4	.01	0.15	0.00–2.81	.91

^a^Severe glomerulosclerosis- Glomerulosclerosis >50% glomeruli.

^b^Severe Interstitial Fibrosis Tubular Atrophy (IFTA)- IFTA >50%.

Factors affecting the patient survival at one year on univariate analysis were advanced age (HR = 1.02, *p* = .04), high serum creatinine (HR = 1.4, *p* = .05) , high anti-GBM titers [increase by 20 IU/ml] (HR = 1.009, *p* = .05) and dialysis dependency at presentation (HR = 1.05, *p* = .01) whereas on multivariate analysis advanced age of the patient (HR = 1.92, *p* = .03), high serum creatinine levels (HR = 1.9, *p* = .04) , and high anti-GBM titers (HR = 1.01, *p* = .03) were associated with poor patient survival (Table 6).

**Table 6.** Showing prognostic factors for patient survival at one year in anti- glomerular basement membrane patients.

	Univariate analysis			Multivariate analysis		
Variable	HR	CI (±95%)	*p* Value	HR	CI (±95%)	*p* Value
Age	1.02	1.00–1.04	.04	1.92	1.86–2.99	.03
Reduced Urinary Output	0.70	0.19–2.54	.06	3.04	0.54–17.00	.2
High Serum Creatinine (mg/dl)	1.47	1.3–2.26	.05	1.9	1.01–3.62	.04
Mean Percentage Of Crescents	1.00	0.97–1.03	.8	1.00	0.94–1.07	.8
^a^Severe Glomerulosclerosis	1.00	0.98–1.03	.5	1.03	0.95–1.10	.4
^b^Severe Anti-GBM Titers	1.00	1.00–1.01	.05	1.01	1.00–1.02	.03
Dialysis Dependency At Presentation	1.05	0.22–4.82	.01	2.94	0.23–37.4	.4
ANCA Positivity	1.03	0.34–3.10	.9	1.31	0.11–15.5	.8

^a^Severe glomerulosclerosis- Glomerulosclerosis >50% glomeruli.

^b^Severe Interstitial Fibrosis Tubular Atrophy (IFTA)- IFTA >50%.

## Discussion

Anti-GBM disease accounted for 0.5% of the total biopsied cases which is in consensus of it being a rare disease. The mean age of presentation was fifth decade in our study which is similar to other studies [15]. However, bimodal distribution of age was not found in our study. This disease has been exteremely uncommon in children [16]. We reported only a single pediatric case of 10 year old boy who achieved complete remission on follow up. The bimodal presentation of this disease has been described with two peaks, one each at 20-39 years and 60-79 years in many studies [4, 17–19]. However few studies from the West have described a single peak at 6–7^th^ decade only [2]. Also, there was no significant difference in the mean age of patients with either single antibody or double positive patients in our study. This is in contrast to previous reports in which double positive patients have been reported to be older than single positive patients [1, 17,18, 20].

The predominant light microscopic pattern was diffuse crescentic pattern, however, mesangial hypercellularity was noted in 16.7% cases and endocapillary proloferation was seen in 10.4% of cases. These changes are a part of morphological spectrum of this disease, which starts with mesangial expansion followed by focal segmental glomerulonephritis, segmental necrosis, and breaks in GBM forming crescent [19].

In our study two patients (4.16%) did not show an increase in serum anti-GBM titers. In the literature about 2–8% of patients with anti-GBM disease are negative for anti-GBM antibody by enzyme immunoassays (ELISA) or Western blot. Moreover, ELISA also shows false positive results. In these patients biopsy still remains a gold standard for diagnosis. Chemiluminescence immunoassay (CIA) is a recent addition to the detection of anti-GBM antibodies. It has a good sensitivity and specificity and is relatively faster than conventional ELISA [21].

Pulmonary renal syndrome due to involvement of both kidneys and lungs was found in 29.1% of total anti-GBM cases in our study. The incidence of pulmonary involvement in anti-GBM disease varies widely in literature ranging from as low as 23% [19] in few studies to as high as 63% in others [22]. Previously the literature reported incidence was between 40–60% [15]. However, recent reports have shown a less incidence of pulmonary-renal syndrome, in only 20–40% of patients [23]. In addition its incidence is further lower in elderly patients (>65 years) as compared to younger cohort [24]. Although pulmonary-renal picture is usually seen in young males and elderly females but in our study most of the patients were males (57.1%) with a mean age of 47 years. When compared to renal limited disease, patients presenting with pulmonary-renal syndrome had a higher frequency of associated hypertension and oliguria. On biopsy these patients had a higher percentage of crescents, increased interstitial inflammation and glomerulosclerosis as compared to the renal limited disease. They also had higher mean anti-GBM titers in their serum and increased rates of dialysis dependency at presentation. These findings suggest that the pulmonary involvement is a sign of severe disease. We could not find such comparisons in the previously described studies. Hirayama K [19] showed that the mean serum creatinine, blood leukocytosis, degree of anemia and urinary protein were higher in Goodpasture syndrome as compared to renal limited disease. We did not replicate such results other than serum creatinine, which was higher in renal-pulmonary involvement. In one of the largest single cohort study from China, patients with renal-pulmonary syndrome had significantly lower hemoglobin levels than renal limited disease. There was, however, no difference in their degree of hypertension, oliguria or serum creatinine levels [17].

Double Antibody Positive disease constituted 29.1% of the cases in our study with a higher frequency of females as compared to single positive (anti-GBM) group. Their biopsy findings also differed from single anti-GBM antibody positive group in terms of chronic changes, glomerulosclerosis and tubular atrophy/interstitial fibrosis, which were more in double positive patients. While similar finding has been reported in some studies [4], others do not show a correlation between the activity/chronicity indices of biopsy and dual positivity [3]. Other observations made in the present study were that the formation of periglomerular granulomas were significantly higher in dual positive group but the mean anti-GBM titers were higher in the single antibody group, cause being unknown. Cui et al. [18] found that the degree of proteinuria and frequency of nephrotic syndrome was higher in ANCA negative patients as compared to double antibody positive cases, the same results were not replicated in our study.

The predictors of ESRD at one year included oliguria at presentation, high serum creatinine levels as well as features of chronicity on biopsy (glomerulosclerosis and IFTA). Coexisting lung hemorrhage was a poor prognostic factor in univariate analysis, however in multivariate analysis it was not statistically significant. Though not extensively studied Alchi et al. [3] also found oliguria to be a strongest predictor of both reduced patient and renal survival. Oliganuria at presentation, severity of renal dysfunction and degree of chronicity on biopsy have been associated with poor renal outcome in other studies as well [1,3,4].

Patient survival in our study was significantly less than the western data [3] (40.4% vs 88%). One of the reasons could be more infection related deaths in immunosuppressed patients in countries like India with poor socio-economic status. Factors that predict poor patient survival in our study was advanced age of the patients, high serum creatinine levels and high anti-GBM titers. Serum creatinine level at presentation was a robust predictor of both renal and patient survival, probably because it correlates well with the degree of renal injury. Advanced age, as an independent predictor of poor patient survival, has been studied by others also [3].

There are conflicting reports in the literature about renal survival of double positive cases as compared to anti-GBM glomerulonephritis. However in our study there was no difference in the survival of coexisting lung involvement as well as double antibody patients.

A number of cases of atypical anti-GBM have been published since its first recognition by Wilson and Dixon [1, 25,26]. We report four cases of atypical anti-GBM in our study with linear staining along GBM on immunofluorescence. Other causes of linear staining on immunofluorescence were ruled out before making a diagnosis of atypical anti-GBM. One of the cases had MPGN pattern on light microscopy without any glomerular crescent formation and presented as nephritic-nephrotic syndrome. Two other cases were of anti-GBM along with IgA nephropathy. The fourth case showed a tubulointerstitial nephritis picture on light microscopy and had low complements. None of them had oliguria, ANA or associated ANCA antibodies. Lung involvement was present only in a single case. Two of the cases had anti-GBM just higher than normal limit, one had mildly elevated levels and fourth case had moderately high anti-GBM titers. The hepatitis and HIV serology were negative in all the cases. All the four patients did not require intensive triple therapy and three of them went in remission. These cases need to be recognized and diagnosed accurately because of the difference in treatment and course of the disease in them. Lesser degree of pulmonary involvement, mild renal insufficiency and better survival of these atypical anti-GBM cases have also been described previously [24]. However, further studies are required to recognize and prognosticate such cases.

**LIMITATION:** The major limitation of the study was that this was a retrospective study and many patients were lost to follow-up.

## Conclusion

Anti-GBM is a rare disease with varied presentations and poor prognosis. Patients with pulmonary-renal syndrome show a severe disease whereas double positive have more of chronic changes on renal biopsy. Besides advanced age, high serum creatinine and anti-GBM antibody levels, chronic changes in renal biopsy also predict poor renal prognosis. So, renal biopsy is mandatory not only for diagnosis but also for prognostication of the disease. While evaluating kidney biopsies of anti-GBM patients, atypical variants, which constitute a small percentage, should also be kept in mind.

## Data Availability

The datasets used and/or analyzed during the current study available from the corresponding author on reasonable request.

## References

[1] McAdoo SP, Pusey CD. Anti-glomerular basement membrane disease. Clin J Am Soc Nephrol. 2017;12(7):1162–1172. 28515156 10.2215/CJN.01380217 PMC5498345

[2] Canney M, O'Hara PV, McEvoy CM, et al. Spatial and temporal clustering of anti-glomerular basement membrane disease. Clin J Am Soc Nephrol. 2016;11(8):1392–1399. 27401523 10.2215/CJN.13591215 PMC4974897

[3] Alchi B, Griffiths M, Sivalingam M, et al. Predictors of renal and patient outcomes in anti-GBM disease: clinicopathologic analysis of a two-centre cohort. Nephrol Dial Transplant. 2015;30:821–828. 25609740 10.1093/ndt/gfu399

[4] Van Daalen EE, Jennette JC, McAdoo SP, et al. Predicting outcome in patients with anti-GBM glomerulonephritis. Clin J Am Soc Nephrol.. 2018;13(1):63–72. 29162595 10.2215/CJN.04290417 PMC5753308

[5] Huart A, Josse AG, Chauveau D, et al. Outcomes of patients with Goodpasture syndrome: a nationwide cohort-based study from the French Society of Hemapheresis. J Autoimmun. 2016;73:24–29. 27267459 10.1016/j.jaut.2016.05.015

[6] Levy JB, Turner AN, Rees AJ, et al. Long-term outcome of anti-glomerular basement membrane antibody disease treated with plasma exchange and immunosuppression. Ann Intern Med. 2001;134(11):1033–1042. 11388816 10.7326/0003-4819-134-11-200106050-00009

[7] Cattran DC, Feehally J, Cook HT, et al. Kidney disease improving global outcomes (KDIGO) glomerulonephritis work group. KDIGO clinical practice guideline for glomerulonephritis. Kidney Int Suppl. 2012;2:139–274.

[8] Rivera F, López-Gómez JM, Pérez-García R. Clinicopathologic correlations of renal pathology in Spain. Kidney Int. 2004;66(3):898–904. 15327378 10.1111/j.1523-1755.2004.00833.x

[9] Greenhall GHB, Salama AD. What is new in the management of rapidly progressive glomerulonephritis? Clin Kidney J. 2015;8(2):143–150. 25815169 10.1093/ckj/sfv008 PMC4370308

[10] Marques C, Carvelli J, Biard L, et al. Prognostic factors in anti-glomerular basement membrane disease: a multicenter study of 119 patients. Front Immunol. 2019;10:1665. 31396214 10.3389/fimmu.2019.01665 PMC6662558

[11] Troxell ML, Houghton DC. Atypical anti-glomerular basement membrane disease. Clin Kidney J. 2016;9(2):211–222. 26985371 10.1093/ckj/sfv140 PMC4792615

[12] Agarwal R. Defining end-stage renal disease in clinical trials: a framework for adjudication. Nephrol Dial Transplant. 2016;31(6):864–867. 26264780 10.1093/ndt/gfv289

[13] Levey AS, Stevens LA, Schmid CH, et al. CKD-EPI (ChronicKidneyDiseaseEpidemiologyCollaboration). A new equation to estimate glomerular filtration rate. Ann Intern Med. 2009;150(9):604–612. 19414839 10.7326/0003-4819-150-9-200905050-00006 PMC2763564

[14] Grabowski B. “P < 0.05” might not mean what you think: American Statistical Association Clarifies P Values. J Natl Cancer Inst. 2016;108(8):djw194. 27510514 10.1093/jnci/djw194 PMC5017929

[15] Jennette JC. Rapidly progressive crescentic glomerulonephritis. Kidney Int. 2003;63(3):1164–1177. 12631105 10.1046/j.1523-1755.2003.00843.x

[16] Williamson SR, Phillips CL, Andreoli SP, et al. A 25-year experience with pediatric anti-glomerular basement membrane disease. Pediatr Nephrol. 2011;26(1):85–91. 20963446 10.1007/s00467-010-1663-2

[17] Cui Z, Zhao J, Jia XY, et al. Anti-glomerular basement membrane disease outcomes of different therapeutic regimens in a large single-center Chinese cohort study. Medicine. 2011;90(5):303–311. 21862934 10.1097/MD.0b013e31822f6f68

[18] Cui Z, Zhao M, Xin G, et al. Characteristics and prognosis of chinese patients with anti-glomerular basement membrane disease. Nephron Clin Pract. 2005;99(2):c49–c55. 15637429 10.1159/000083133

[19] Hirayama K, Yamagata K, Kobayashi M, et al. Anti-glomerular basement membrane antibody disease in Japan: part of the nationwide rapidly progressive glomerulonephritis survey in Japan. Clin Exp Nephrol. 2008;12(5):339–347. 18392773 10.1007/s10157-008-0051-8

[20] Levy JB, Hammad T, Coulthart A, et al. Clinical features and outcome of patients with both ANCA and anti-GBM antibodies. Kidney Int. 2004;66(4):1535–1540. 15458448 10.1111/j.1523-1755.2004.00917.x

[21] Tan Y, Pang W, Jia X, et al. Comparison of the performance of a chemiluminescence assay and an ELISA for detection of anti-GBM antibodies. Ren Fail. 2020;42(1):48–53. 31885301 10.1080/0886022X.2019.1702056 PMC6968565

[22] Imperio VL, Ajello E, Pieruzzi F, et al. Clinicopathological characteristics of typical and atypical anti-glomerular basement membrane nephritis. J Nephrol. 2017;30(4):503–509. 28382508 10.1007/s40620-017-0394-x

[23] Segelmark M, Hellmark T, Wieslander J. The prognostic significance in Goodpasture's disease of specificity, titre and affinity of anti-glomerular-basement-membrane antibodies. Nephron Clin Pract. 2003;94(3):c59–c68. 12902632 10.1159/000072022

[24] Cui Z, Zhao J, Jia XY, et al. Clinical features and outcomes of anti-glomerular basement membrane disease in older patients. Am J Kidney Dis. 2011;57(4):575–582. 21168945 10.1053/j.ajkd.2010.09.022

[25] Nasr SH, Collins AB, Alexander MP, et al. The clinicopathologic characteristics and outcome of atypical anti-glomerular basement membrane nephritis. Kidney Int. 2016;89(4):897–908. 26994577 10.1016/j.kint.2016.02.001

[26] Yao S, Chen M, Liu Y. Atypical anti-glomerular basement membrane disease with IgA nephropathy: a case report. Int J ClinExp Med. 2017;10(11):15611–15614.

